# Supplementary material for: Same law, diverging practice: Comparative analysis of Endangered Species Act consultations by two federal agencies
Source: PLoS One. 2020 Mar 20;15(3):e0230477. doi: 10.1371/journal.pone.0230477 (PMC7083319; doi:10.1371/journal.pone.0230477)
Supplement: S1 Fig — (DOCX) [file pone.0230477.s001.docx]

**SI FIGURE 1: INFORMAL STICKER CONCURRENCE**

**
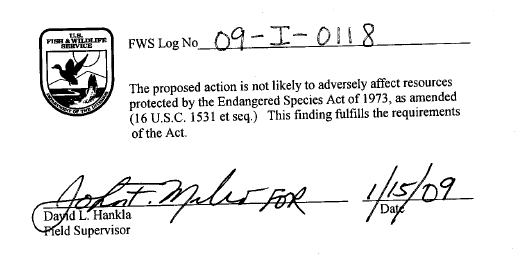
**

Complete informal consultation included in Open Science Framework archive at https://dx.doi.org/10.17605/OSF.IO/KAJUQ. Note that there is no accompanying analysis to clarify why this informal consultation was found not likely to adversely affect the species or any listed critical habitat.
