# Supplementary material for: Same law, diverging practice: Comparative analysis of Endangered Species Act consultations by two federal agencies
Source: PLoS One. 2020 Mar 20;15(3):e0230477. doi: 10.1371/journal.pone.0230477 (PMC7083319; doi:10.1371/journal.pone.0230477)
Supplement: S1 Appendix — (DOCX) [file pone.0230477.s002.docx]

**SI APPENDIX 1: SCORING RUBRIC FOR FORMAL ESA SECTION 7 CONSULTATIONS**

**Environmental Baseline Completeness (Total Points: 5)**

- - 1. Does the Environmental Baseline address the status of the species in the action area? (1)
    2. Is there a mention of past/ongoing threats to the species in the action area? (1)
    3. Does the Environmental Baseline take past consultations in the action area into consideration? (1)
    4. Is there mention of critical habitat (or lack thereof) for the species? Does said critical habitat overlap with the action area? (1)
    5. Does the baseline include State, tribal, local and private actions already affecting the species that will occur contemporaneously with the consultation in progress, as per the handbook? (1)

**Effects of the Action Completeness (Total Points: 2)**

1. There is a clear and defined cause and effect analysis of the action. (1)
2. The consultation gives an explanation as to if and how said action will negatively affect sea turtles. (1)

**Species Status Completeness (Total Points: 5)**

1. Does the consultation adequately describe the species and its habitat/critical habitat? (1)
2. Is the life history of the species addressed? (1)
3. Is there a detailed demographic analysis (if available for the species), including population size, variability and stability? (1)
4. Is the status and distribution of the species addressed, including reasons for listing? (1)
5. Is there an analysis of the species/critical habitat likely to be affected by the action? (1)

**Cumulative Effects Completeness (Total Points: 2)**

1. Does the consultation consider the likelihood of the species to be able to recover? (1)
2. Does the consultation consider the effects of *future* State, tribal, local or private actions that are reasonably certain to occur, as per the handbook? (1)
