# Supplementary material for: Same law, diverging practice: Comparative analysis of Endangered Species Act consultations by two federal agencies
Source: PLoS One. 2020 Mar 20;15(3):e0230477. doi: 10.1371/journal.pone.0230477 (PMC7083319; doi:10.1371/journal.pone.0230477)
Supplement: S2 Appendix — (DOCX) [file pone.0230477.s003.docx]

**SI APPENDIX 2: SCORING RUBRIC FOR INFORMAL ESA SECTION 7 CONSULTATIONS**

**Informal Criteria Baseline (Total Points: 5)**

- - 1. Mentions the action (1)
    2. Some analysis of the action (1)
    3. Some analysis of the impacted species (1)
    4. Reason the consultation stayed informal is mentioned (1)
    5. Map of the area affected by the action (1)
