# Supplementary material for: Same law, diverging practice: Comparative analysis of Endangered Species Act consultations by two federal agencies
Source: PLoS One. 2020 Mar 20;15(3):e0230477. doi: 10.1371/journal.pone.0230477 (PMC7083319; doi:10.1371/journal.pone.0230477)
Supplement: S3 Appendix — (DOCX) [file pone.0230477.s004.docx]

**SI APPENDIX 3: CONSULTATION PROCESS QUESTIONS FOR FISH AND WILDLIFE SERVICE AND NATIONAL MARINE FISHERIES SERVICE BIOLOGISTS**

1. Can you tell me a bit about how the consultation process usually begins for you?
2. How frequently do you work on consultation? Has this number increased or decreased in recent years? Why might that be so?
3. How common is it to ask the action agency to provide more information on the action?
4. Have you seen a change over time in the way consultations are completed?
5. The number of consultations for FWS in Florida has been steadily decreasing since 2008 (according to the TAILS database there were 1099 in 2008 vs. 347 in 2014). Do you have an impression of how often you aren’t consulted on things?
6. Is there a consultation key for sea turtles, similar to the FWS Wood Stork Consultation Key? If not, is this something the Service would consider doing? Would this be an improvement to the process? Would you be in favor of a more standardized way to approach the consultation process? (Keys, a standardized ITP, etc.)
7. Can you explain the process of going through the literature and files on hand to satisfy the “best possible science” condition?
8. How do you exercise precaution when dealing with scientific uncertainty surrounding the effects of an action on a species/critical habitat? How much benefit of the doubt do you give to the species? Does it differ depending on the situation? Is this an issue you deal with on a regular basis?
9. How much time do you spend on the average consultation? FWS TAILS database says the average days for approval for formal consultations is 89 (13 for informal) days. Does that seem right?
10. Is pervious take ever tallied (formally or informally) to get a sense of how much has been done to a species over time? In your view, would this be a feasible/helpful thing to implement?
11. How often do you consult the section 7 Handbook?
12. Do you ever get requests for re-initiation of consultations?
13. NMFS is taking the lead on the revision of the handbook this year. What would you like to see in the revision? In your opinion, is there something that should be clarified?
14. What is your opinion on making all of the final documents publicly available (NMFS has PCTS, Vero Beach has the formal consultations online but not the informal documents)?
15. Where is there the most room for improvement in the consultation process? Does it work well as is?
